# Tryptophan Intake and Tryptophan Losses in Hemodialysis Patients: A Balance Study

**DOI:** 10.3390/nu11122851

**Published:** 2019-11-21

**Authors:** Adrian Post, Marleen Huberts, Enya Poppe, Martijn van Faassen, Ido P. Kema, Steffie Vogels, Johanna M. Geleijnse, Ralf Westerhuis, Karin J. R. Ipema, Stephan J. L. Bakker, Casper F. M. Franssen

**Affiliations:** 1 Department of Nephrology, University Medical Center Groningen; University of Groningen, 9713 GZ Groningen, The Netherlands; marleenhuberts@gmail.com (M.H.); enya.poppe@gmail.com (E.P.); k.ipema@umcg.nl (K.J.R.I.); s.j.l.bakker@umcg.nl (S.J.L.B.); c.f.m.franssen@umcg.nl (C.F.M.F.); 2 Department of Laboratory Medicine, University Medical Center Groningen; University of Groningen, 9713 GZ Groningen, The Netherlands; h.j.r.van.faassen@umcg.nl (M.v.F.); i.p.kema@umcg.nl (I.P.K.); 3 Dialysis Center Groningen, 9713 GZ Groningen, The Netherlands; S.Vogels@mzh.nl (S.V.); r.westerhuis@dcg.nl (R.W.); 4 Department of Human Nutrition and Health, Wageningen University, 6708 PB Wageningen, The Netherlands; marianne.geleijnse@wur.nl

**Keywords:** dialysis, dietary diaries, excretion, tryptophan, kynurenine, hydroxyindoleacetic acid

## Abstract

Tryptophan depletion is common in hemodialysis patients. The cause of this depletion remains largely unknown, but reduced nutritional tryptophan intake, losses during dialysis or an increased catabolism due to an inflammatory state are likely contributors. Currently, little is known about tryptophan homeostasis in hemodialysis patients. We assessed dietary tryptophan intake, measured plasma tryptophan during dialysis, and measured the combined urinary and dialysate excretion of tryptophan in 40 hemodialysis patients (66 ± 15 years and 68% male). Patients had low tryptophan concentrations (27 ± 9 µmol/L) before dialysis. Mean dietary tryptophan intake was 4454 ± 1149 µmol/24 h. Mean urinary tryptophan excretion was 15.0 ± 12.3 μmol/24 h, dialysate excretion was 209 ± 67 μmol/24 h and combined excretion was 219 ± 66 µmol/24 h, indicating only 5% of dietary tryptophan intake was excreted. No associations were found between plasma tryptophan concentration and tryptophan intake, plasma kynurenine/tryptophan ratio or inflammatory markers. During dialysis, mean plasma tryptophan concentration increased 16% to 31 ± 8 µmol/L. Intradialytic increase in plasma tryptophan was associated with a lower risk of mortality, independent of age, sex and dialysis vintage (HR: 0.87 [0.76–0.99]; *P* = 0.04). Tryptophan intake was well above the dietary recommendations and, although tryptophan was removed during dialysis, mean plasma tryptophan increased during dialysis. The cause of this phenomenon is unknown, but it appears to be protective.

## 1. Introduction

Depression is common among dialysis patients [1] and is known to adversely affect patients’ quality of life and is associated with higher rates of both hospitalization and mortality [2,3,4,5,6,7]. The causes of depression are multifactorial, consisting of both psychosocial and biological factors [3,8,9]. A potentially modifiable biological factor is tryptophan deficiency [10,11,12,13]. In dialysis patients, lower plasma tryptophan concentrations have been reported, with an accumulation of tryptophan metabolites [14,15,16,17,18]. The causes of the low plasma tryptophan concentrations remain largely unknown, but reduced dietary tryptophan intake, losses during dialysis or an increased catabolism due to an inflammatory state are likely contributors [19,20,21,22]. However, to date, little is known about tryptophan homeostasis during hemodialysis, as no studies exist that have measured tryptophan intake, plasma changes and excretion simultaneously.

In this study, we assessed both dietary tryptophan intake, plasma tryptophan before and after dialysis and the urinary and dialysate excretion rate of tryptophan. With these data we are able to (1) assess plasma tryptophan concentration and changes in plasma tryptophan concentration during hemodialysis; (2) assess dietary intake of tryptophan in hemodialysis patients; (3) investigate how much tryptophan is excreted during dialysis and by urinary excretion in patients with residual diuresis; (4) determine whether dietary tryptophan intake, indoleamine 2,3-dioxygenase activity (IDO) and inflammatory markers are determinants of plasma tryptophan concentration before dialysis; and (5) investigate whether plasma tryptophan concentration before dialysis or the intradialytic change in plasma tryptophan concentration is associated with survival in hemodialysis patients. 

## 2. Materials and Methods

### 2.1. Design and Study Population

This observational study was performed according to ethical standards laid down in the 1964 Declaration of Helsinki and its later amendments, and was approved by the Medical Ethical Committee of the University Medical Center Groningen, The Netherlands. All participating patients gave written informed consent. Inclusion criteria were twice or thrice weekly hemodialysis with 3–5 h per treatment, a dialysis vintage of ≥2 months and absence of clinical signs of infection. As the nature of the study was observational, no control group was included.

Most patients dialyzed three times per week, on either Monday–Wednesday–Friday or on Tuesday–Thursday–Saturday. In both cases, the mid-week dialysis session was used in this study. For patients dialyzing twice weekly, the last dialysis session of the week was used. Hypertension was defined as predialysis systolic blood pressure >140 mmHg and/or diastolic blood pressure >90 mmHg. A history of diabetes and cardiovascular disease was obtained from the patients’ medical records. Cardiovascular disease was defined as a history of ischemic heart disease, congestive heart failure, coronary artery bypass grafting, percutaneous coronary intervention, stroke, or peripheral vascular disease. Blood pressure and weight were measured before and after hemodialysis. Body surface area (BSA) was calculated using the formula of Du Bois and Du Bois [23]. Handgrip strength was measured using the JAMAR hydraulic hand dynamometer (Lafayette instrument company, Lafayette, IN, USA) before the HD session during which dialysate was collected. Measurements were performed at the non-fistula arm, which was the dominant arm in most patients. In patients without an arteriovenous fistula, handgrip strength was measured at the dominant hand.

### 2.2. Dialysis Settings

All studies were performed with the Fresenius 5008 dialysis apparatus with a low-flux dialyzer (Fresenius Medical Care, Bad Homburg, Germany) using smartbag dialysate concentrations (Fresenius Medical Care). Blood flow and dialysate flow were between 200 and 300 mL/min and between 500 and 700 mL/min, respectively. Dialysate temperature was 36.0 or 36.5 °C. Dialysis fluid sodium varied from 136 to 140 mmol/L, potassium from 1 to 3 mmol/L, depending on the plasma potassium concentration, calcium from 1.25 to 1.50 mmol/L and bicarbonate from 34 to 38 mmol/L.

### 2.3. Sample Collection

During the dialysis session, all dialysate was collected in a 200-liter tank. The total dialysate volume was measured by calculating the weight difference of the tank before and after the hemodialysis session. At the end of the dialysis, homogenous samples were taken from the dialysate for analysis [24]. Blood was drawn directly from the dialysis line, at the start of hemodialysis and five minutes before the end of the dialysis session. Patients with significant residual diuresis, defined as a urine production of more than 200 mL/24 h, were asked to collect two 24 h urine collections before the hemodialysis session, during which the dialysate was collected. For patients with a thrice weekly dialysis schedule this was the complete interdialytic urine production.

### 2.4. Laboratory Measurements

Plasma, urine and dialysate tryptophan, kynurenine and hydroxyindoleacetic acid (5-HIAA) were measured using a validated liquid chromatography tandem mass spectrometry method [25]. Reference intervals for this analytical method were previously determined: 45.5–83.1 μmol/L for plasma tryptophan, 1.14–3.02 μmol/L for plasma kynurenine and 20.1–52.6 nmol/μmol for the kynurenine to tryptophan ratio [25]. The kynurenine to tryptophan ratio was used as a surrogate marker for IDO activity. Sodium, potassium, urea, and total protein were measured on Roche routine chemistry analyzers (Modular P/Cobas C, Roche Diagnostics, Mannheim, Germany). Other laboratory measurements were performed with automated and validated routine methods (Roche Diagnostics, Mannheim, Germany). Routinely measured equilibrated Kt/V values (Daugirdas equation [26]), and protein catabolic rates were extracted from the patients’ medical records.

To determine the combined excretion rate of tryptophan, kynurenine and 5-HIAA, we combined the dialysate excretion rate with the urinary excretion rate, if available [24]. The combined excretion rate of tryptophan, kynurenine and 5-HIAA was calculated as follows:

Combined excretion rate of × (µmol/24 h) = (V_Dialysate_ × D_x_ × *n*)/7 + UE_x_ (1)

in which V_Dialysate_ = total volume of the spent dialysate (L); D_x_ = measured tryptophan, kynurenine or 5-HIAA concentration in the collected dialysate (µmol/L); *n* = number of dialysis sessions per week; UE_x_ = 24 h urinary excretion (µmol/24 h) of tryptophan, kynurenine or 5-HIAA, averaged from two 24 h urine collections. 

To calculate urinary and dialysate clearances of tryptophan, kynurenine and 5-HIAA, the excretion rates were related to the plasma concentrations over the interdialytic interval. The exact course of tryptophan, kynurenine and 5-HIAA throughout the dialysis session is not known. In this study, we used the mean of plasma concentrations before and after dialysis:

Urinary clearance of × (mL/min) = UE_x_/((P_x before_ + P_x after_)/2) × (1000/1440) (2)

Dialysate clearance of × (mL/min) = ((V_Dialysate_ × D_x_ × *n*)/7)/((P_x before_ + P_x after_)/2) × (1000/1440) (3)

in which UE_x_ = 24 h urinary excretion (µmol/24 h) of tryptophan, kynurenine or 5-HIAA, averaged from two 24 h urine collections; P_x before_ = Plasma concentration before dialysis of tryptophan, kynurenine or 5-HIAA (µmol/L); P_x after_ = Plasma concentration after dialysis of tryptophan, kynurenine or 5-HIAA (µmol/L); D_x_ = measured tryptophan, kynurenine or 5-HIAA concentration in the collected dialysate (µmol/L); *n* = number of dialysis sessions per week.

The fractional reabsorption of tryptophan, kynurenine and 5-HIAA was defined as 1 minus the fractional excretion of tryptophan, kynurenine or 5-HIAA, which was calculated by dividing the urinary clearance of tryptophan, kynurenine or 5-HIAA by the urinary clearance of creatinine [27].

### 2.5. Dietary Intake Assessment

Participating patients were asked to record all their food and fluid intake for a period of 5 days, starting 5 days before the dialysis session of interest. The number of servings was expressed in natural units (e.g., slice of bread or apple) or household measures (e.g., cup or spoon). The dietary diaries were self-administered and filled out at home. The diaries were processed using Dutch food tables to calculate the tryptophan composition of the food. 

### 2.6. Statistical Analysis

Data analyses and computations were performed with SPSS 24.0 software (IBM, Armonk, NY, USA), R version 3.5.1 software (The R-Foundation for Statistical Computing, Vienna, Austria) and GraphPad Prism version 6 (GraphPad software, San Diego, CA, USA). Baseline data are presented as mean ± standard deviation for normally distributed data, as median (interquartile range) for non-normally distributed data, and as numbers (percentages) for nominal data. A two-sided *P* < 0.05 was considered to indicate statistical significance. Differences between values before and after dialysis were tested with a paired sample *t*-test. Cross-sectional associations of plasma tryptophan concentration before dialysis and the intradialytic change in plasma tryptophan concentration with baseline variables were studied using linear regression models. Regression coefficients were given as standardized beta values, referring to the number of standard deviations a dependent variable changes per standard deviation increase of the independent variable, thereby allowing for comparison of the strength of the associations of different variables. Linear regression coefficients were displayed for univariate and multivariate models. In multivariate models adjustments were made for the potential confounders age, sex, body mass index (BMI) and dialysis vintage.

Cox regression analyses were employed to investigate the association of plasma tryptophan concentration before dialysis and intradialytic plasma tryptophan increase with all-cause mortality. The continuous surveillance system of the outpatient program ensured that there was up-to-date information on patient status. Endpoints were recorded until May 2019 by a qualified researcher, with no loss to follow-up. Due to the relatively small number of events, Cox regression models were built in a stepwise fashion. Adjustments were made for age and sex (model 2), with additional adjustments for either dialysis vintage (model 3), BMI (model 4), plasma iron (model 5), plasma kynurenine (model 6), equilibrated Kt/V (model 7), protein catabolic rate (model 8) and plasma albumin (model 9). Age, sex, BMI, dialysis vintage, equilibrated Kt/V, protein catabolic rate and plasma albumin were a priori selected potential confounders. Plasma iron and plasma kynurenine were selected based on their strong associations in linear regression analyses. 

## 3. Results

### 3.1. Patient Characteristics

Plasma, urine and dialysate tryptophan, kynurenine and 5-HIAA were determined in 40 hemodialysis patients, 68% of which were male. An overview of patient characteristics is shown in Table 1. Mean age at inclusion was 66 ± 15 years with a median dialysis vintage of 17 (11–48) months. Nearly all patients (37 out of 40) were dialyzed thrice weekly, three patients were dialyzed twice weekly. Dialysis duration was 4 hours per session in 32 patients, while it was 3 to 3.5 hours per session in three patients and 4.5 to 5 h per session in five patients. A total number of 27 out of 40 patients had residual diuresis, with a mean urinary volume of 0.97 ± 0.66 liters per day. Mean BMI and BSA were 25.2 ± 4.5 kg/m^2^ and 1.95 ± 0.24 m^2^, respectively. Hypertension, diabetes and cardiovascular disease were prevalent in 58%, 23% and 30% of the patients, respectively. Differences in baseline characteristics between patients with and without dietary diaries are shown in Table S1. Except for a difference in the proportion of Caucasians (100% versus 79% in patients with and those without available 5-day dietary diaries, respectively; *P* = 0.04), baseline characteristics were similar amongst both groups (*P* > 0.05). 

### 3.2. Clinical and Laboratory Parameters Before and After Dialysis

An overview of clinical and laboratory parameters before and after dialysis is shown in Table 2. During dialysis, plasma tryptophan increased from 27.0 ± 9.0 µmol/L to 31 ± 8.2 µmol/L (*P* = 0.001). In contrast, plasma kynurenine decreased from 5.1 ± 2.3 µmol/L to 3.0 ± 0.9 µmol/L (*P* < 0.001) and plasma 5-HIAA decreased from 1.38 ± 0.72 µmol/L to 0.53 ± 0.30 µmol/L (*P* < 0.001). 

**Figure 1:**
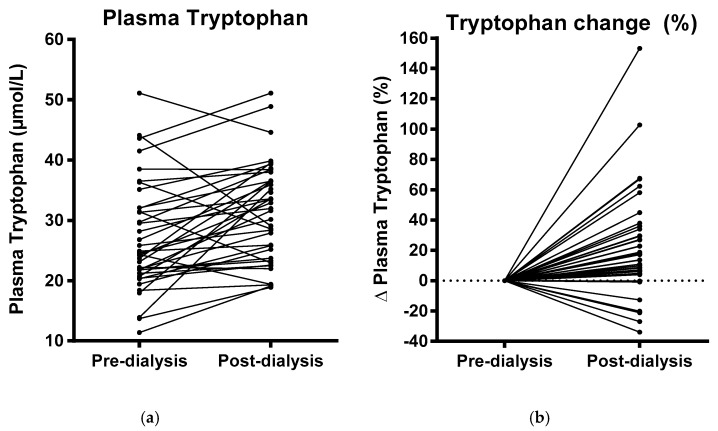
Plasma tryptophan concentrations before and after dialysis (**a**) and relative plasma tryptophan change during dialysis (**b**).

In line with slight hemoconcentration, occurring due to fluid removal during dialysis, plasma hemoglobin (7.0 ± 0.6 vs. 7.5 ± 0.9 mmol/L; *P* = 0.001), hematocrit (0.35 ± 0.03 vs. 0.37 ± 0.04; *P* = 0.003) and albumin (38 ± 5 vs. 42 ± 6; *P* < 0.001) increased during dialysis. Consistent with this, mean body weight declined from 80.0 ± 18.0 kg to 78.7 ± 17.8 kg, and mean ultrafiltration volume was 1876 ± 903 mL. Plasma urea (19 ± 5 vs. 6 ± 2 mmol/L; *P* < 0.001) and creatinine (691 ± 207 vs. 257 ± 95 µmol/L; *P* < 0.001) decreased during dialysis. Systolic blood pressure (145 ± 22 vs. 141 ± 28 mmHg; *P* = 0.18) and diastolic blood pressure (69 ± 12 vs. 68 ± 12 mmHg; *P* = 0.09) declined somewhat, albeit not significantly. A graphical representation of the absolute and relative change in plasma tryptophan during dialysis is shown in Figure 1.

### 3.3. Tryptophan Intake and Excretion

Dietary diaries were completed by 26 out of 40 patients. Mean dietary tryptophan intake was 4454 ± 1149 µmol/24 h, ranging from 2740 µmol/24 h to 7429 µmol/24 h. In milligrams per 24 h, this corresponds to a mean intake of 909 ± 235 mg/24 h, ranging from 560 mg/24 h to 1517 mg/24 h. All patients had a dietary intake higher than the dietary tryptophan recommendation of 250–425 mg/24 h [28]. An overview of intake and excretion is shown in Table 3.

Mean urinary excretion rate of tryptophan, kynurenine and 5-HIAA in the whole cohort was 15.0 ± 12.3 µmol/24 h, 3.1 ± 2.4 µmol/24 h and 12.3 ± 7.4 µmol/24 h, respectively. Mean urinary excretion rate of tryptophan, kynurenine and 5-HIAA of the 26 patients that also completed 5-day dietary diaries was 12.8 ± 10.6 µmol/24 h, 3.0 ± 2.4 µmol/24 h and 11.5 ± 5.4 µmol/24 h, respectively. Urinary clearance of tryptophan, kynurenine and 5-HIAA in the whole cohort was 0.25 ± 0.21 mL/min, 0.33 ± 0.24 mL/min and 8.5 ± 8.4 mL/min, respectively. Urinary clearance of tryptophan, kynurenine and 5-HIAA in patients that also completed 5-day dietary diaries was 0.22 ± 0.19 mL/min, 0.31 ± 0.21 mL/min and 8.0 ± 4.7 mL/min, respectively. Fractional reabsorption of tryptophan, kynurenine and 5-HIAA in the whole cohort was 95 ± 4%, 94 ± 4% and −36 ± 60%, respectively. Fractional reabsorption of tryptophan, kynurenine and 5-HIAA in patients that also completed 5-day dietary diaries was 96 ± 3%, 94 ± 3% and −39 ± 53%, respectively.

In the whole cohort, tryptophan, kynurenine and 5-HIAA removal during the dialysis of interest was 499 ± 146 µmol, 74 ± 33 µmol and 23 ± 11 µmol, respectively. In patients that also completed 5-day dietary diaries, tryptophan, kynurenine and 5-HIAA removal was 492 ± 148 µmol/, 67 ± 19 µmol and 21 ± 9 µmol, respectively. Dialysate excretion rate of tryptophan, kynurenine and 5-HIAA in the whole cohort was 209 ± 67 µmol/24 h, 31 ± 15 µmol/24 h and 10 ± 5 µmol/24 h, respectively. Dialysate excretion rate in patients that also completed 5-day dietary diaries was 208 ± 65 µmol/24 h, 28 ± 8 µmol/24 h and 9 ± 4 µmol/24 h, respectively. Dialysate clearance of tryptophan, kynurenine and 5-HIAA in the whole cohort was 3.5 ± 1.0 mL/min, 3.3 ± 0.8 ml/min and 4.3 ± 1.1 mL/min, respectively. Dialysate clearance of tryptophan, kynurenine and 5-HIAA in patients that also completed 5-day dietary diaries was 3.5 ± 0.9 mL/min, 3.2 ± 0.8 mL/min and 4.1 ± 1.0 mL/min, respectively. 

The combined excretion rate of tryptophan, kynurenine and 5-HIAA of the whole cohort was 219 ± 66 µmol/24 h, 33 ± 15 µmol/24 h and 18 ± 7 µmol/24 h, respectively. Combined excretion rate of tryptophan, kynurenine and 5-HIAA in patients that also completed 5-day dietary diaries was 218 ± 68 µmol/24 h, 31 ± 9 µmol/24 h and 17 ± 5 µmol/24 h, respectively. Compared to the dietary tryptophan intake, only 4.9% of tryptophan was excreted unchanged. The combined excretion rate of the metabolites kynurenine and 5-HIAA accounted for 0.7% and 0.4% of the dietary tryptophan intake, respectively. 

### 3.4. Linear Regression Analyses

Results of linear regression analyses are shown in Table 4. Univariately, plasma tryptophan concentration before dialysis was positively associated with plasma iron (β = 0.420; *P* = 0.009), plasma kynurenine (β = 0.745; *P* < 0.001), tryptophan excretion (β = 0.588; *P* < 0.001) and kynurenine excretion (β = 0.531; *P* = 0.001). Multivariate analyses revealed independent associations of plasma tryptophan concentration before dialysis with plasma albumin (β = 0.359; *P* = 0.04) and the amount of hours dialysis per week (β = 0.355; *P* = 0.03). Multivariate analyses strengthened the association of plasma tryptophan concentration before dialysis with plasma iron (β = 0.451; *P* = 0.009), tryptophan excretion (β = 0.738; *P* < 0.001) and kynurenine excretion (β = 0.576; *P* < 0.001), while the association with plasma kynurenine became slightly weaker (β = 0.698; *P* < 0.001). 

Univariately, intradialytic plasma tryptophan increase was associated with creatinine excretion (β = 0.335; *P* = 0.04), plasma iron (β = −0.361; *P* = 0.03), plasma creatinine (β = 0.368; *P* = 0.02), plasma kynurenine (β= −0.530; *P* = 0.001), kynurenine excretion (β = −0.380; *P* = 0.02) and 5-HIAA excretion (β = 0.316; *P* = 0.05). Multivariate analyses revealed an independent association between intradialytic plasma tryptophan increase and the amount of hours dialysis per week (β = −0.319; *P* = 0.05). Multivariate analyses strengthened the associations of intradialytic plasma tryptophan increase with plasma iron (β = −0.386; *P* = 0.03) and kynurenine excretion (β = −0.445; *P* = 0.006), while weakening the association with plasma kynurenine (β = −0.488; *P* = 0.002). Creatinine excretion (β = 0.159; *P* = 0.17), plasma creatinine (β = 0.259; *P* = 0.09) and 5-HIAA excretion (β = 0.285; *P* = 0.09) were no longer associated with intradialytic plasma tryptophan increase in the multivariate model. 

### 3.5. All-Cause Mortality

During a median follow-up of 3.0 (2.9–3.5) years, eight patients (21%) died. Patients who died had comparable plasma tryptophan concentrations to patients who survived (27.3 ± 8.6 versus 26.9 ± 9.2 µmol/L; *P* = 0.92). Patients who died had a mean plasma tryptophan decrease of 0.4 ± 8.3 µmol/L during dialysis, while patients who survived had a mean plasma tryptophan increase of 5.6 ± 6.4 µmol/L during dialysis (*P* = 0.04). Prospective analyses on the association of plasma tryptophan before dialysis and intradialytic plasma tryptophan increase with all-cause mortality are shown in Table 5. In Cox regression analyses, plasma tryptophan before dialysis was not associated with all-cause mortality (HR: 1.00 [0.93–1.08]; *P* = 0.98). After adjustment for potential confounders, the association remained not significant. In contrast, intradialytic plasma tryptophan increase was associated with improved survival (HR: 0.91 [0.83–0.99]; *P* = 0.04). Adjustment for age and sex did not materially affect the association of intradialytic plasma tryptophan increase with all-cause mortality (HR: 0.87 [0.76–0.99]; *P* = 0.04). Further adjustment for potential confounders (model 3–8) did not materially change the association. However, after adjusting for plasma albumin, the association of intradialytic plasma tryptophan increase with all-cause mortality lost significance. 

## 4. Discussion

To the best of our knowledge, this is the first study that comprehensively assessed tryptophan in both diet, plasma, urine and dialysate. Plasma tryptophan concentrations in hemodialysis patients were lower than in healthy individuals, and during dialysis plasma tryptophan concentrations increased in most hemodialysis patients. Dietary intake of tryptophan was higher than the recommended dietary tryptophan intake for the general population in each of the hemodialysis patients. Only 5% of this dietary tryptophan intake was excreted in urine and dialysate. Dietary tryptophan intake, the plasma kynurenine/tryptophan ratio (reflecting IDO activity) and inflammatory markers were not associated with lower plasma tryptophan values. Plasma tryptophan was not associated with survival, while an intradialytic plasma tryptophan increase was associated with a lower risk of mortality.

Tryptophan is an essential amino acid required for protein synthesis, however, compared to the other amino acids it is generally found in low amounts in proteins and plasma [29,30]. In addition to protein synthesis, tryptophan is also an obligatory substance for the production of a variety of bioactive molecules, including serotonin and niacin [30]. The diversity of physiological functions and the low body content of tryptophan increases the potential risk for disturbances in tryptophan homeostasis [29,31], such as low plasma tryptophan concentrations. These low plasma tryptophan concentrations may not be without risk, as a recent study showed that low plasma tryptophan concentrations are associated with a higher risk of mortality in patients with cardiovascular disease [32]. In patients on hemodialysis, previous studies have found that plasma tryptophan concentrations are decreased compared to healthy controls [17,18]. In our study, plasma tryptophan concentrations in dialysis patients were also lower than those reported for healthy individuals in other studies [18]. A possible reason for decreased plasma tryptophan concentrations could be an insufficient intake of tryptophan, as dialysis patients often suffer from loss of appetite and malnutrition [22,33]. However, in our study we found that mean dietary tryptophan intake, assessed through 5-day dietary diaries, was 4454 ± 1149 µmol/24 h. This corresponds to a dietary intake of 909 ± 235 mg/24 h, which is well above the recommended dietary intake of 250–425 mg/24 h [28,34]. In fact, the dietary tryptophan intake in each patient was above this recommendation. The dietary tryptophan intakes of the hemodialysis patients in this study were in line with findings from the general population, e.g., 826 ± 3 mg/24 h tryptophan in the NHANES 2001–2012 study [35]. It is thus highly unlikely that the observed low plasma tryptophan concentrations in hemodialysis patients are caused by insufficient tryptophan intake.

We found that both plasma kynurenine concentrations and the plasma kynurenine to tryptophan ratio, reflecting IDO activity, were high compared to reference values based on 120 health individuals [25]. Previous studies have reported that an inflammatory state may cause the upregulation of IDO activity and lead to increased tryptophan catabolism via the kynurenine pathway, causing the tryptophan depletion. However, in this study we found neither a significant association of plasma tryptophan with IDO activity, nor with hs-CRP or interleukin-6 [17]. 

Based on its small molecular size (204.2 g/mol), tryptophan is expected to be removed during hemodialysis. Indeed, we found tryptophan in the dialysate of each dialysis patient and mean excretion per dialysis session was 499 ± 146 µmol. When combined with urinary excretion and calculated back to daily excretion, mean excretion rate was 219 ± 66 µmol/24 h. Tryptophan excretion accounts for roughly 5% of daily dietary intake, indicating that the majority of the ingested tryptophan is metabolized before being excreted. In line with the previous literature, we found that tryptophan and kynurenine undergo extensive tubular reabsorption in patients that still urinate, while 5-HIAA is actively secreted by the kidneys [36,37]. The active secretion of 5-HIAA explains the high urinary clearance of 5-HIAA and why urinary 5-HIAA excretion contributes the most to the combined excretion rate. 

Despite the fact that the amount of tryptophan removed during a dialysis session is nearly 20 times higher than the plasma tryptophan values before dialysis, in 82% of the patients, plasma tryptophan did not decrease during dialysis. On the contrary, mean tryptophan increased from 27.0 ± 9.0 µmol/L to 31.3 ± 8.2 µmol/L. Percentage-wise, we found a mean increase of 16%, which is more than the increase of compounds not able to cross the dialysis membrane, i.e., plasma albumin (11%) and hemoglobin (7%). The combination of significant losses in dialysis and an increase larger than can be explained by hemoconcentration implies that there must be a release of tryptophan into the systemic circulation during dialysis. Possibilities could be release from proteolysis in muscles or increased gastro-intestinal release. Unfortunately, we were unable to further investigate the mechanisms of this increase. We did, however, attempt to investigate whether the change in plasma tryptophan during dialysis can be considered a beneficial mechanism or the opposite. In both univariate and multivariate Cox regression, the intradialytic change in plasma tryptophan during dialysis was significantly associated with improved survival, with a hazard ratio consistent with intradialytic increase of plasma tryptophan being beneficial. Interestingly, the association remained significant after adjustment for age, sex, dialysis vintage, equilibrated Kt/V and protein catabolic rate, but lost significance after adjusting for plasma albumin. However, no associations were found between intradialytic plasma tryptophan increase and predialysis plasma albumin, nor was there an interaction between intradialytic plasma tryptophan increase and predialysis plasma albumin in the association with mortality. These findings indicate that plasma albumin is a more important determinant for survival in dialysis than the intradialytic changes in plasma tryptophan. 

The strength of this study is that we collected the total dialysate instead of taking several samples during the dialysis sessions, thereby increasing the accuracy of the dialysate excretion measurements. Also, dietary diaries were recorded for 5-days, decreasing possible bias due to day-to-day variability in food intake. However, we acknowledge that our study has limitations. Although this is the largest study to date on tryptophan balance in hemodialysis patients, the sample size was relatively small. In addition, due to the observational nature of this study we were unable to determine the mechanism of the intradialytic plasma tryptophan increase. During dialysis sessions, patients were given a light snack and 2–3 drinks. Unfortunately, we did not measure the tryptophan content of the dietary intake during the dialysis session. In addition, we only measured two of the many tryptophan metabolites. We also did not study the degree to which dietary tryptophan is actually absorbed in the gut, a process in which the microbiome may play a role. Furthermore, because our participants were Caucasian, our results cannot be extrapolated to other ethnic groups.

## 5. Conclusions

In this study we found that (1) plasma tryptophan concentrations in hemodialysis patients were lower than in healthy individuals and, during dialysis, mean plasma tryptophan concentrations increased; (2) dietary intake of tryptophan was well above the dietary tryptophan recommendation; (3) only 5% of the dietary tryptophan intake was excreted in urine and dialysate; (4) dietary tryptophan intake, IDO activity and inflammatory markers were not associated with the lower plasma tryptophan values and (5) an increase in plasma tryptophan during hemodialysis was associated with a lower risk of mortality. The results of this study indicate that the low tryptophan values in hemodialysis patients cannot fully be attributed to low dietary intake, high IDO activity or an inflammatory state. Despite being a small molecule that crosses the dialysis membrane, plasma tryptophan concentrations increased during dialysis. While this study shows that the intradialytic increase in plasma tryptophan may be a protective mechanism, further research is required to explore the mechanisms underlying both the low plasma tryptophan values and the intradialytic plasma tryptophan increase.

## Figures and Tables

**Table 1:** Patient characteristics.

Baseline Characteristics	Average/Number
**Demographics**	
Age, years	66 ± 15
Gender, *n* male (%)	27 (68)
Race, *n* Caucasian (%)	37 (93)
**Dialysis-related**	
Dialysis sessions, *n* (%)	
Two sessions per week	3 (8)
Three sessions per week	37 (93)
Hours per dialysis, *n* (%)	
3 to 3.5 h	3 (8)
4 h	32 (80)
4.5 to 5 h	5 (13)
Residual diuresis, *n* (%)	27 (68)
Urinary volume, L	0.97 ± 0.66
Dialysis vintage, months	17 (11–48)
Ultrafiltration volume, mL	1876 ± 903
Equilibrated Kt/V per dialysis	1.27 ± 0.45
Protein catabolic rate, g/kg/24 h	1.08 ± 0.31
**Body composition**	
Target body weight, kg	80.8 ± 18.5
Interdialytic weight gain, kg	1.12 ± 1.14
Height, m	1.76 ± 0.10
BMI, kg/m^2^	25.2 ± 4.5
BSA, m^2^	1.95 ± 0.24
**Pre-existing disease**	
Hypertension, *n* (%)	23 (58)
Diabetes, *n* (%)	9 (23)
Cardiovascular disease, *n* (%)	12 (30)

**Table 2:** Laboratory measurements and clinical measurement before and after dialysis.

Variable	Before Dialysis	After Dialysis	Change	*P*-Value
**Laboratory measurements**				
Tryptophan, µmol/L	27.0 ± 9.0	31.3 ± 8.2	+16%	0.001
Kynurenine, µmol/L	5.1 ± 2.3	3.0 ± 0.9	−41%	<0.001
5-HIAA, µmol/L	1.38 ± 0.72	0.53 ± 0.30	−62%	<0.001
Kynurenine/tryptophan ratio	0.19 ± 0.06	0.10 ± 0.03	−47%	<0.001
Hemoglobin, mmol/L	7.0 ± 0.6	7.5 ± 0.9	+7%	0.001
Hematocrit, v/v	0.35 ± 0.03	0.37 ± 0.04	+6%	0.003
Albumin, g/L	38 ± 5	42 ± 6	+11%	<0.001
Urea, mmol/L	19 ± 5	6 ± 2	−68%	<0.001
Creatinine, µmol/L	691 ± 207	257 ± 95	−63%	<0.001
**Clinical measurements**				
Systolic blood pressure, mmHg	145 ± 22	141 ± 28	−3%	0.18
Diastolic blood pressure, mmHg	69 ± 12	68 ± 12	−1%	0.09
Pulse, min^−1^	73 ± 13	71 ± 19	−3%	0.36
Body weight, kg	80.0 ± 18.0	78.7 ± 17.8	−2%	<0.001

**Table 3:** Intakes and excretions in the whole cohort and a subgroup who completed dietary diaries.

Variable	Whole Cohort (*n* = 40)	Subgroup with Dietary Intake (*n* = 26)	Percentage of Dietary Intake *
**Intake**			
Dietary tryptophan intake, µmol/24 h		4454 ± 1149	
Dietary energy intake, kCal/24 h		1869 ± 423	
Dietary protein intake, g/24 h		75 ± 22	
**Urinary removal**			
**Urinary excretion rate**			
Tryptophan excretion, µmol/24 h	15.0 ± 12.3	12.8 ± 10.6	0.3%
Kynurenine, µmol/24 h	3.1 ± 2.4	3.0 ± 2.4	0.1%
5-HIAA, µmol/24 h	12.3 ± 7.4	11.5 ± 5.4	0.3%
**Urinary clearance**			
Tryptophan excretion, mL/min	0.25 ± 0.21	0.22 ± 0.19	
Kynurenine, mL/min	0.33 ± 0.24	0.31 ± 0.21	
5-HIAA, mL/min	8.5 ± 8.4	8.0 ± 4.7	
**Fractional reabsorption**			
Tryptophan excretion, %	95 ± 4	96 ± 3	
Kynurenine, %	94 ± 4	94 ± 3	
5-HIAA, %	−36 ± 60	−39 ± 53	
**Dialysate removal**			
**Single dialysis removal**			
Tryptophan excretion, µmol/dialysis	499 ± 146	492 ± 148	
Kynurenine, µmol/dialysis	74 ± 33	67 ± 19	
5-HIAA, µmol/dialysis	23 ± 11	21 ± 9	
**Dialysate excretion rate**			
Tryptophan excretion, µmol/24 h	209 ± 67	208 ± 65	4.7%
Kynurenine, µmol/24 h	31 ± 15	28 ± 8	0.6%
5-HIAA, µmol/24 h	10 ± 5	9 ± 4	0.2%
**Dialysate clearance**			
Tryptophan excretion, mL/min	3.5 ± 1.0	3.5 ± 0.9	
Kynurenine, mL/min	3.3 ± 0.8	3.2 ± 0.8	
5-HIAA, mL/min	4.3 ± 1.1	4.1 ± 1.0	
**Combined removal**			
**Combined excretion rate**			
Tryptophan excretion, µmol/24 h	219 ± 66	218 ± 68	4.9%
Kynurenine, µmol/24 h	33 ± 15	31 ± 9	0.7%
5-HIAA, µmol/24 h	18 ± 7	17 ± 5	0.4%

* Calculated as excretion/dietary tryptophan intake * 100%.

**Table 4:** Linear regression analyses of plasma tryptophan before dialysis and the absolute plasma tryptophan increase during dialysis.

	Plasma Tryptophan				Intradialytic Plasma Tryptophan Increase			
	Univariate		Multivariate		Univariate		Multivariate	
Dependent Variables	Std. β	*P*-Value	Std. β	*P*-Value	Std. β	*P*-Value	Std. β	*P*-Value
**Demographics**								
Age, years	0.204	0.22	0.240	0.16	−0.117	0.48	−0.140	0.42
Sex, *n* male (%)	0.135	0.42	0.183	0.28	−0.103	0.54	−0.121	0.48
BMI, kg/m^2^	−0.144	0.39	−0.113	0.53	0.217	0.19	0.188	0.28
BSA, m^2^	−0.217	0.19	−0.038	0.68	0.107	0.52	−0.100	0.27
**Dialysis**								
Dialysis vintage, months	−0.018	0.91	−0.020	0.91	0.110	0.51	0.106	0.56
Dialysis per week, hours	0.276	0.09	0.355	**0.03**	−0.241	0.15	−0.319	**0.05**
Ultrafiltration, ml/day	−0.040	0.82	0.128	0.50	0.274	0.12	0.179	0.35
Equilibrated Kt/V per dialysis	0.161	0.33	0.138	0.42	−0.180	0.28	−0.189	0.26
Protein catabolic rate, g/kg/24 h	0.265	0.11	0.253	0.15	0.034	0.84	0.063	0.72
**Muscle**								
Handgrip strength, kg	−0.302	0.07	−0.158	0.27	0.246	0.14	0.172	0.22
Creatinine excretion, mmol/24 h	−0.227	0.17	−0.006	0.96	0.335	**0.04**	0.159	0.17
**Blood pressure**								
Systolic blood pressure, mmHg	−0.097	0.56	−0.052	0.76	0.059	0.73	0.034	0.84
Diastolic blood pressure, mmHg	−0.162	0.33	−0.119	0.50	0.222	0.18	0.239	0.17
**Laboratory measurements**								
Hemoglobin, mmol/L	0.217	0.19	0.218	0.22	0.140	0.40	0.154	0.39
Hematocrit, v/v	0.256	0.12	0.180	0.30	0.059	0.73	0.169	0.33
Plasma sodium, mmol/L	−0.050	0.77	−0.066	0.72	0.058	0.73	0.087	0.64
Plasma potassium, mmol/L	−0.093	0.58	−0.088	0.63	0.149	0.37	0.156	0.38
Plasma iron, µmol/L	0.420	**0.009**	0.451	**0.009**	−0.361	**0.03**	−0.386	**0.03**
Plasma albumin, g/L	0.312	0.06	0.359	**0.04**	0.208	0.21	0.213	0.24
Plasma urea, mmol/L	0.035	0.83	0.038	0.81	0.231	0.16	0.280	0.07
Plasma creatinine, µmol/L	−0.275	0.10	−0.162	0.30	0.368	**0.02**	0.259	0.09
**Inflammatory markers**								
Hs-CRP, mg/L	−0.027	0.87	−0.084	0.64	−0.205	0.22	−0.215	0.21
Il-6, pg/mL	−0.061	0.72	−0.139	0.41	−0.231	0.16	−0.228	0.17
**Tryptophan homeostasis**								
Dietary Tryptophan intake, µmol/24 h	0.129	0.53	0.378	0.07	0.212	0.30	0.085	0.71
Plasma kynurenine, µmol/L	0.745	**<0.001**	0.698	**<0.001**	−0.530	**0.001**	−0.488	**0.002**
Plasma 5-HIAA, µmol/L	0.013	0.94	−0.107	0.49	0.009	0.96	0.060	0.70
Kynurenine/tryptophan ratio	0.069	0.68	−0.002	0.99	−0.234	0.16	−0.206	0.23
Tryptophan excretion, µmol/24 h	0.588	**<0.001**	0.738	**<0.001**	−0.119	0.48	−0.250	0.13
Kynurenine excretion, µmol/24 h	0.531	**0.001**	0.576	**<0.001**	−0.380	**0.02**	−0.445	**0.006**
5-HIAA excretion, µmol/24 h	−0.022	0.89	0.021	0.91	0.316	**0.05**	0.285	0.09

The independent variables in the analyses are log_2_ transformed plasma tryptophan before dialysis (µmol/L) or absolute intradialytic increase in plasma tryptophan concentration (µmol/L). Regression coefficients are given standardized beta values, the latter referring to the number of standard deviations a dependent variable changes, per standard deviation increase of the independent variable. Significant associations are indicated in bold. Univariate: crude, either plasma tryptophan or intradialytic tryptophan increase. Multivariate: adjusted for age, sex, BMI and dialysis vintage. Abbreviations: BMI, body mass index; BSA, body surface area; Std. β, standardized beta.

**Table 5:** Cox regression analyses of plasma tryptophan and intradialytic plasma tryptophan increase.

	Plasma Tryptophan		Intradialytic Tryptophan Increase	
Model	HR (95% CI)	*P*-Value	HR (95% CI)	*P*-Value
Model 1	1.00 (0.93–1.08)	0.98	0.91 (0.83–0.99)	0.04
Model 2	0.97 (0.87–1.07)	0.54	0.87 (0.76–0.99)	0.04
Model 3	0.97 (0.87–1.07)	0.53	0.87 (0.76–0.99)	0.04
Model 4	0.97 (0.87–1.07)	0.53	0.87 (0.75–0.99)	0.04
Model 5	0.93 (0.81–1.07)	0.31	0.86 (0.75–0.99)	0.03
Model 6	0.93 (0.82–1.05)	0.23	0.86 (0.74–1.00)	0.05
Model 7	0.97 (0.87–1.07)	0.51	0.84 (0.73–0.98)	0.02
Model 8	0.98 (0.88–1.09)	0.73	0.86 (0.75–0.99)	0.04
Model 9	1.00 (0.91–1.10)	0.96	0.94 (0.84–1.07)	0.36

Model 1: crude, either plasma tryptophan or intradialytic tryptophan increase; model 2: adjusted for age and sex; model 3: as model 2, additionally adjusted for dialysis vintage; model 4: as model 2, additionally adjusted for BMI; model 5: as model 2, additionally adjusted for plasma iron; model 6: as model 2, additionally adjusted for plasma kynurenine; model 7: as model 2, additionally adjusted for equilibrated Kt/V; model 8, as model 2, additionally adjusted for protein catabolic rate; model 9, as model 2, additionally adjusted for plasma albumin. Abbreviations: HR, hazard ratio; CI, confidence interval.

## Supplementary Materials

The following are available online at https://www.mdpi.com/2072-6643/11/12/2851/s1, Table S1: Baseline characteristics amongst patients with and without dietary diaries available.
