# Supplementary material for: Tryptophan Intake and Tryptophan Losses in Hemodialysis Patients: A Balance Study
Source: Nutrients. 2019 Nov 21;11(12):2851. doi: 10.3390/nu11122851 (PMC6950375; doi:10.3390/nu11122851)
Supplement: Supplementary file 1 [file nutrients-11-02851-s001.pdf]

Supplementary data

Table S1. Baseline characteristics amongst patients with and without dietary diaries available.

Baseline characteristics	Whole cohort (n=40)	Patients with dietary diary (n=26)	Patients without dietary diary (n=14)	P for difference
**Demographics**				
Age, years	66 ± 15	64 ± 15	69 ± 17	0.30
Gender, n male (%)	27 (68)	15 (58)	12 (86)	0.07
Race, n Caucasian (%)	37 (93)	26 (100)	11 (79)	0.04
**Dialysis-related**				
Dialysis sessions, n (%)				
2 sessions per week	3 (8)	1 (4)	2 (14)	0.28
3 sessions per week	37 (93)	25 (96)	12 (86)	
Hours per dialysis, n (%)				
3 to 3.5 hours	3 (8)	3 (11)	0 (0)	0.19
4 hours	32 (80)	21 (81)	11 (79)	0.87
4.5 to 5 hours	5 (13)	2 (8)	3 (2)	0.21
Residual diuresis, n (%)	27 (68)	19 (73)	8 (57)	0.48
Urinary volume, L	0.97 ± 0.66	0.89 ± 0.61	1.15 ± 0.79	0.36
Dialysis vintage, months	17 [11-48]	19 [8-53]	16 [13-47]	0.94
Ultrafiltration volume, ml	1876 ± 903	1793 ± 894	2040 ± 936	0.45
Equilibrated Kt/V per dialysis	1.27 ± 0.45	1.29 ± 0.53	1.23 ± 0.26	0.71
Protein catabolic ratio, g/kg/24 h	1.08 ± 0.31	1.08 ± 0.34	1.08 ± 0.24	0.97
**Body composition**				
Target body weight, kg	80.8 ± 18.5	81.6 ± 18.9	79.4 ± 18.5	0.76
Interdialytic weight gain, kg	1.12 ± 1.14	1.00 ± 1.08	1.33 ± 1.26	0.41
Height, m	1.76 ± 0.10	1.76 ± 0.10	1.76 ± 0.10	0.92
BMI, kg/m^2	25.2 ± 4.5	25.2 ± 4.5	25.2 ± 4.7	0.99
BSA, m^2	1.95 ± 0.24	1.95 ± 0.26	1.94 ± 0.21	0.87
**Pre-existing disease**				
Hypertension, n (%)	23 (58)	15 (58)	8 (57)	0.99
Diabetes, n (%)	9 (23)	7 (27)	2 (14)	0.36
Cardiovascular disease, n (%)	12 (30)	6 (23)	6 (43)	0.19

P for difference was assessed using independent sample *t*-test, Mann-Whitney *U* test and Chi-Squared test.
Abbreviations: BMI, body mass index; BSA, body surface area.
